# Instituting solidarity: the role of research institutes in advancing global health equity

**DOI:** 10.1186/s12961-026-01500-6

**Published:** 2026-06-19

**Authors:** M. Ndu, H. Healey, J. Natukunda, U. Karunakara, C. Atuire, E. Nouvet

**Affiliations:** 1 Faculty of Health Sciences, School of Health Studies, Western University, London, Ontario Canada; 2 Centre for Tropical Medicine and Global Health, University of Oxford, Oxford, England; 3 College of Humanities, Department of Philosophy and Classics, University of Ghana, Accra, Ghana; 4 Global Health Justice Partnership, Yale University, New Haven, Connecticut United States of America

**Keywords:** Global health, Solidarity, Research institutes, Qualitative research

## Abstract

**Background:**

Despite the increasing discourse around equity, social justice and partnership, global health (GH) research remains shaped by entrenched asymmetries in funding, authorship, data ownership and priority-setting. Solidarity, framed variously as a moral principle or political stance, has been promoted as both corrective and a necessary response to these inequities. Yet, the operationalization of solidarity within research institutes (RIs) (i.e. how leaders interpret, institutionalize and negotiate solidaristic commitments) remains underexplored. This paper draws on findings from a global study of meanings and practices relevant to solidarity among diverse GH stakeholders.

**Methods:**

In 2024–2025, we conducted exploratory qualitative research using semistructured interviews (*N* = 75) with a range of GH stakeholders. In this paper, we draw on the 13 interviews conducted with leaders of GH RIs to highlight findings specific to solidarity in and for GH research. Data analysis was conducted using team-based thematic analysis supported by NVIVO 15 (QSR).

**Results:**

Thematic analysis of interview transcripts identified five themes in research institute leaders’ accounts of how solidarity is or is not enactment in their GH research experience. Each theme describes a requirement or outcome of solidarity-informed research, and includes that such research: (1) generates tangible equitable results or outcomes; (2) accounts for how relationships and impacts unfold over time; (3) operationalizes and depends on less hierarchical ways of working; (4) demands fair distribution of burdens and benefits in research; and (5) requires deep, active listening.

**Conclusions:**

This paper provides insight on how solidarity is understood and can be actualized in GH research practice, according to directors in GH RIs who occupy critical roles in structuring and legitimizing GH research agendas. The findings emphasize the need to treat solidarity in and for GH research not as a static ethical ideal, but as a dynamic, relational practice requiring reflexivity, structural reforms and sustained commitment. RIs, as ethical actors, must reconfigure their practices to meaningfully institutionalize solidarity through co-design, authorship, partnership and funding practices. Ultimately, this paper contributes to a growing agenda to reimagine GH research not merely as a technical endeavour but also as a site of political and ethical action.

**Supplementary Information:**

The online version contains Supplementary Material available at https://doi.org/10.1186/s12961-026-01500-6.

## Introduction

To work in global health (GH) is to work within projects and programmes that have a stated purpose to improve health and well-being. Such projects are routinely framed as responding to and committed to reducing inequities [1, 2]. Discourse within the sector has long invoked the need for transformations to the current distribution of morbidity and mortality risks, and access to opportunities for living a dignified and fulfilling life. In the past 10 years, there has been a proliferation of calls for substantive change to the structures and systems underpinning GH funding, projects and programmes [3–5]. The current GH system needs reform, to walk the walk of its commitments to equity [6, 7]. The sector remains steeped in disparities that reflect and buttress colonial-era inequities between the Global North and Global South: in terms of access to and control over funding, conditions and criteria for the production of knowledge deemed legitimate to inform policy, authorship, priority-setting and data ownership [8–11]. Alongside calls for reform, significant work has been conducted to define ethical and equitable GH partnerships and research [12–27].

Solidarity has emerged more recently within calls for needed transformation to the GH sector [28–30]. In the literature, solidarity has been conceptualized in several ways. Some scholars have framed solidarity in and for GH as an ethical principle grounded in shared humanity and moral responsibility [28, 31–33]. Others have framed it as a political act of resistance against historical and ongoing structural inequities [34–36]. Another influential account describes solidarity as an enacted commitment to bear certain costs to assist others with whom one recognizes a relevant connection [37, 38]. Others still have drawn attention to the gaps between stated aspirations or commitments to solidarity in response to GH inequities and crises, and what transpires in action [39–42]. For instance, the COVID-19 Vaccines Global Access (COVAX) facility was created to ensure fair and global access to coronavirus disease 2019 (COVID-19) vaccines, with an ambitious goal of delivering 2 billion doses by the end of 2021 to safeguard vulnerable groups in lower-income nations [43, 44]. In reality, it achieved only a fraction of this aim, distributing fewer than half the planned doses and covering less than 5% of worldwide vaccinations [45, 46].

Solidarity’s meanings and potential as an organizing principle for ethical and equity-advancing action in GH, it could be argued, has been muddied by such wide and inconsistent usage [47]. What centring or augmenting solidarity in and for GH implies in practice remains, for the most part, unclear. With respect to GH research, one author group has provided more specifics, describing solidarity as involving commitments to co-production, equitable authorship, capacity strengthening and the redistribution of power and resources [48]. Whether or not these definitions hold across research settings remains to be confirmed through further inquiry.

Should solidarity be an organizing principle for GH work? If yes, what does this imply or involve in practice? Since 2023, our team has been exploring these questions through a number of methods and as part of a 5-year study entitled Moving beyond Solidarity Rhetoric in GH. As part of this project, we conducted interviews (*N* = 75) with diverse GH stakeholders to gain insight on meanings of solidarity and its implications for practice. These interviews took the word solidarity, rather than a definition of solidarity, as their point of departure. Participants were not provided with a definition of solidarity. Instead, they were asked what the term meant to them in their work. They were asked to discuss what it includes, what it looks like when successful or unsuccessful, and why it matters. One category of interviews conducted was with leaders working in GH research institutes (RIs).

In this paper, we present an analysis of these interviews with GH research institute (RI) leaders. More specifically, we present key themes in GH RI leaders’ descriptions of what solidarity in action involves, in the context of GH research. RIs and their leaders are approached here as important sites for the (re)formation of power (in)equities in the sector. RIs play a pivotal role in shaping the norms, practices and agendas of GH research. As gatekeepers of knowledge production, they occupy a unique position to either reinforce or challenge systemic inequalities [27, 49, 50]. Their governance structures, internal practices and engagement with specific research settings and participants shape the way ethical commitments in GH and equitable partnerships in research are institutionalized or performed. This analysis responds to a critical gap in the literature, moving beyond calls for (more) solidarity in GH that can be vague or unclear in terms of their implication for practice. Understanding what these leaders recognize as solidarity in GH RIs serves to clarify meanings and practical implications of solidarity commitments for others engaged in GH research.

We use solidarity commitments here to reference deliberate actions made to stand together in mutual support in pursuit of justice, equality and/or shared goals. These actions go beyond passive agreements and involve active participation in the well-being of others. In this paper, we examined solidaristic relationships that structure GH research practice, including among researchers and institutions, within global research partnerships, and between researchers and the communities and populations affected by GH inequities. If the meanings and practices of solidarity surfaced here do not resonate with readers, this article may, alternatively, usefully fuel discussion and debate on what solidarity is, should be and can achieve in and for GH research.

Therefore, in this paper we examine how leaders of GH RIs interpret and describe the enactment of solidarity in GH research practice.

## Methods

### Study design

The larger study, Meanings and Practices of Solidarity in and for Global Health [51], from which this subset of interviews is drawn for analysis, used a qualitative research design using in-depth, one-on-one semistructured interviews. The study conducted interviews with 75 participants across three groups: civil society organizations (CSOs), RIs and GH influencers. In this article, we focus on a subset of 13 interviews with actors situated in or around RIs working in GH. The 13 participants were specifically selected for their self-identification as GH researchers and strategic roles in shaping their institution’s research approaches to collaboration and partnership. The broader study’s methodological approach is detailed in the published methodology paper [51].

### Sampling and recruitment

Participants were purposefully sampled using maximum variation [52, 53] to ensure diversity across geography, institutional affiliation and professional roles within the research ecosystem. Recruitment combined publicly available information (e.g. institutional websites and staff directories) with network referrals through professional contacts, a common and appropriate strategy in qualitative research for reaching participants with relevant expertise [54]. Where possible, participants were encouraged to share study information with colleagues and partners. In line with the broader study’s protocol, particular attention was given to recruiting individuals from diverse institutional and regional contexts, including those directly engaged in collaborative research. This approach prioritized diversity of perspective and positionality rather than seeking saturation [55], ensuring that the analysis could reflect variations in how solidarity is experienced and enacted across different settings. The target sample for this subset was approximately 15 participants; 13 RI leaders ultimately accepted the invitation to participate. While modest in number, this sample is consistent with qualitative research guidance that emphasizes the value of depth, diversity and contextual variation over numerical size [56, 57]. The analytic sample was considered adequate in relation to the study’s focused aim. The participants’ specialized leadership roles within GH RIs positioned them to provide information-rich accounts relevant to the research aim. This approach is consistent with the concept of information power in qualitative research [58]; the specificity of the sample and the depth of the interview material supported analytic sufficiency within this group. Sample size was determined iteratively using a data adequacy approach. Data adequacy can be assessed through multiple concepts, including information power, conceptual depth and theoretical sufficiency, all of which contribute to establishing sufficiency of the data for meeting the study’s scope and aims [59, 60]. Guided by these principles, alongside feasibility and pragmatic considerations [59, 60], our team collectively assessed when sampling could appropriately conclude across each participant subgroup, including RI. Three team members (J.N., M.N. and E.N.) evaluated data adequacy through ongoing discussion after each interview. These assessments considered three criteria: (1) conceptual depth (whether interviews produced rich, detailed insights from participants with relevant experience); (2) theoretical sufficiency (whether participants provided illustrative examples that support transparent interpretation); and (3) information power (whether the focused study aim, specificity of the sample and the depth of analysis indicate sufficient information had been generated). On the basis of these assessments, we determined that adequate information had been obtained from the 13 interviews.

### Data collection

There were three subsets of interview guides, each designed to be specific to a particular study group. The interview guide for this subset of interviews is attached as Supplementary Material A.1. Each interview lasted between 45 min and 130 min (average 50 min) and were conducted across WHO regions. For the RI subgroup reported here, the interviews were designed to stimulate participants’ retrospective reflections on practices, challenges and aspirations of solidarity in GH research. In using a semistructured interview guide, participants could flexibly define and exemplify solidarity in their own words, while comparability was maintained across the interviews. As such, in the interview guide, no predefined definition of solidarity was included to allow participants to interpret and describe it on the basis of their professional experiences in GH research. Interviews were conducted via Zoom to accommodate participants across different time zones and geographical locations. They were conducted by research team members in either English, Spanish or French, according to the participants’ language preferences, and transcribed in a cleaned, verbatim form (with duplicate words and filler repetitions removed) in the original languages. Spanish and French interviews were subsequently translated into English by fluent language speakers, either through professional services or by research team members, depending on capacity and availability. Transcripts were audited for accuracy by H.H. and then de-identified before analysis.

### Data analysis

The analysis approach followed Braun and Clarke’s [61–64] reflexive thematic analysis, employing an iterative, collaborative coding process and structured codebook that evolved during analysis. Three team members (J.N., M.N. and E.N.) independently coded an initial sample of three transcripts to identify recurring themes and clarify conceptual distinctions. The team then met to review interpretations and refine the codebook, ensuring shared understanding and consistency. The final codebook was applied to all 13 transcripts using NVIVO 15 (QSR) qualitative data analysis software. Primary coding was conducted by two researchers (J.N. and M.N.). An early career researcher (H.H.) and the principal investigator (E.N.) audited the codebook to ensure coding reliability, resolve discrepancies and maintain analytical rigour. When there were differences in coding or interpretation, the team held meetings to reconcile any discrepancies until we reached consensus. Rather than treating disagreement between coders as a problem to resolve, we approached it as analytically productive: a signal of genuine complexity in the data worth exploring. When conflicts arose, we returned to the data together, grounding our discussions in the material itself alongside reflexive practices such as memoing and journaling. This process allowed us to deepen and nuance the themes, arriving at a final thematic structure that meaningfully incorporated each team member’s interpretations. This iterative process ensured the validity and coherence of the final thematic structure. Coding captured anticipated themes (e.g. power asymmetries) and emergent patterns (e.g. performative solidarity), while remaining reflexive to the evolving data. Specifically, coding captured (a) participants’ general understandings and practices of solidarity in and for GH and (b) research-specific examples, practices and concerns, such as collaboration, funding and leadership. Analysis focused on areas of both divergence and convergence across participant responses, with particular attention to the contextual meanings and implications of solidarity in different settings.

### Researchers’ positionality

Our research team composition informed the interpretations in this paper. The core analysis team consisted of two foreign-trained African researchers based in the United Kingdom and Canada, and two Canadian researchers. Our shared commitment to equity-oriented research informed the critical lens through which we explored solidarity in GH research. While our differing affiliations and lived experiences enabled us to reflect on the power asymmetries often present in GH research, we were also attentive to the tensions participants raised in their discussions. We viewed our positionalities not as limitations but as valuable analytical assets. All interviews were conducted by M.N., a foreign-trained African researcher based in Canada, and E.N., a Canadian, both affiliated with a high-income-country institution. None of the participants were previously known to M.N. and E.N. Rapport was established within each interview rather than through prior professional relationships. We recognize that the interviewer’s institutional affiliation, geographic location and positioning within the global health research landscape likely shaped recruitment, interview rapport and the candour with which participants discussed tensions related to power, hierarchy and equity. Participants may have framed accounts of solidarity differently with an interviewer perceived as partially embedded within global health research networks than they would have with someone positioned outside these spaces. We remained attentive to these dynamics throughout data interpretation and analysis. Analysis was shaped by researchers’ positionalities, as meaning-making and knowledge production are always contextual and situated [62]. Our findings reflect our process of analysis, which involved prolonged immersion in data, continuous reflexive practice and a sustained commitment to centring participants’ voices [62, 63].

## Results

Our findings draw on 13 purposively selected interviews with leaders of research. Of the 13 participants, 7 worked in RIs in five low- and middle-income countries (LMICs). The countries from which participants were currently working can be found in Table 1. Among participants, there were many shared understandings of solidarity. The accounts often referenced collaboration between multiple research stakeholders, including those with unequal resources and power. The data presented here confirmed findings from the broader study, namely that solidarity was frequently used interchangeably with other concepts such as collaboration and partnerships. Participants also understood solidarity as involving varying degrees of commitment to equity and social justice. This nuance in understanding suggests that while solidarity can be invoked as a unifying principle, it also carries inherent tensions. These tensions are more visible particularly when resource imbalances or competing priorities are at play and are further explored in the themes presented below. Participants were anonymized using codes (e.g. R11, R28) to preserve confidentiality. The letter “R” refers to research institute participant, while the number represents the interview order.

**Table 1:** Country classification by WHO region

Country	WHO region
Cameroon	African Region (AFR)
India	South-East Asia Region (SEAR)
Argentina	Region of the Americas (AMR)
The Netherlands	European Region (EUR)
Italy	European Region (EUR)
Ghana	African Region (AFR)
Mongolia	Western Pacific Region (WPR)
United States (US)	Region of the Americas (AMR)
Canada	Region of the Americas (AMR)
United Arab Emirates (UAE)	Eastern Mediterranean Region (EMR)

GH RI leaders described successes and challenges in enacting what they understood as solidarity within GH research. Their narratives and reflections illuminated the multiple ways in which solidarity was practised – through tangible research outcomes, the quality of relationships and daily research practices. From these insights, we identified five interconnected themes. Solidarity-informed research: (1) generates tangible equitable results or outcomes; (2) accounts for how relationships and impacts unfold over time; (3) operationalizes and depends on less hierarchical ways of working; (4) demands fair distribution of burdens and benefits in research; and (5) requires deep, active listening. Each theme provides a point of departure for further discussion on what enacting solidarity in GH research may imply or require. In what follows, we provide further detail on each of these themes with supporting quotes from interviews, flagging, as appropriate, connections between the themes as we proceed.

### Theme 1: generates tangible equitable results or outcomes

Participants understood solidarity as meaningful and authentic when research priorities expanded or contributed to concrete structural change, such as more equitable access to health (increased access to medicines, vaccines and other interventions) and amplified community awareness and understanding of their right to health and well-being.

Several leaders expressed concern that solidarity commitments in research risk becoming more symbolic than substantive. As the following participant noted, solidarity without action was too common. Frequent solidarity promises with no corresponding action reduced solidarity to a mere so-called token:

The main one would be that I wouldn’t want it to be a token, like a token of solidarity, and then it starts being used all the time. And I see in my field, a lot of commitments that are not followed by actions. And I’m very scared that, as solidarity or equity, however you want to call it, is becoming a topic that stakeholders are not using for the right reasons. And I think that in some cases, it is the case at the moment. I want to see real ambitious commitments that are followed up with actions. (R28, EUR)

Another participant suggested that direct change within multinational companies is unlikely. For this participant, these multinational corporations engage in health philanthropy such as funding disease eradication campaigns; however, they often do so in pursuit of branded projects to enhance their reputation. For this participant, research can operate as a catalyst for moral awareness, capable of influencing public values and priorities through a bottom-up process wherein the social uptake of research indirectly reshapes economic and political interests:

I cannot change Google now. And I cannot change Facebook. These are multinational companies. It will take you ages to change this. But when I change the people who will care about, let’s say, the climate crisis, the GH issues, etc., Google will take care of that. Not because they are convinced of our point, but because they will lose clients. So, they care about their clients. If the clients tend to have more ethical awareness, moral awareness, and so on, these big companies will care about this. Politicians will care about this because they want to get their votes and so on. Research, leading to increased moral awareness, is the solution. If we think about quick solutions, this is the solution. Effective solutions, this is the solution. Doing something else, I don’t see this succeeding within the timeframe I have. (R052, EMR)

While the above statement draws attention to the complexity of factors, actors and levels of actions that may be involved to effect structural change, research leader perceptions of their work’s impacts were primarily described with reference to how projects and programmes in which they were involved translated into observable and measurable changes in the lives of those most affected by health inequities. Statements such as the following underlined this message:

Then the question for me, and even when I work with my stakeholders, is always, how impactful is this? So, you could say, you’re coming up with all these initiatives, but what does that mean to the people on the ground? Does it reach the people who want to get this medicine? With the vaccines that you’re giving, are they reaching the people who need them? (R32, EUR)

### Theme 2: accounts for how relationships and impacts unfold over time

Building on the previous theme, participants emphasized that solidarity-informed research must also consider how relationships and impacts unfold over time. Participants described sustainability as an essential feature of solidarity in GH research, something that extends beyond project timelines or funding cycles. They understood solidarity as requiring ongoing, action-oriented commitments whose effects endure after external support ends. For many, sustainability depended on community ownership, local leadership and planning for continuity through scaling up successful initiatives or transferring responsibility to local partners. As one participant (R11) emphasized, solidarity loses meaning when confined to short-term actions; instead, it must be sustained through continuous improvement and long-term engagement to achieve lasting impact.

When we talk about solidarity, we have to think about sustainability. At the very least, we need to maintain what we’re doing so that there’s always a continuous improvement. I think that if there’s only one-off solidarity, at some point it won’t have the impact we want. With solidarity, it’s all about continuous improvement. So for me, if a funder comes in today, gives all the money needed for an issue there’s a problem if we haven’t planned how we’re going to move on to the next step, how we’re going to scale up or how we’re going to get the community to take ownership of what they’ve got, and how are they going to take ownership of what they’ve got so that the solidarity action becomes sustainable. (R11, AFR)

As another participant noted, projects lacking strong involvement from local communities or relevant groups often fail to produce enduring results. Without inclusive participation, the participant argued, health inequities will continue to worsen over time rather than improve.

We limit the involvement of local participants or our communities, and then when we see the projects failing, we wonder why. I thought in several cases, and we have done other studies that have shown, and several people have done that. If you have these projects that have very little input from local communities, you will not see success. You may see success probably briefly, but it is not the success that will be sustained over time. If we exclude all the groups that need to be on board, then many times we have these worsening health inequities and we struggle to reduce them. (R43, AFR)

While many participants described the importance of involving communities in the research process as crucial to sustaining project results, several also noted frictions between these understandings and goals of solidarity and reality. A participant pointed out that the increasing focus of funders on demonstrable impact has encouraged researchers to consider the usefulness of their work to communities. However, this participant noted that few funders offer resources for building long-term relationships, even though such co-designed approaches are important for creating research solidarity.

[T]here’s still very few funders that will give you any money to spend time just developing a relationship with the community and building a relationship of trust with the community so that they are willing to work with you. But where that does happen over the course of years and something is genuinely co-designed, then I think there probably is an element of solidarity in there. (R14, SEAR)

As suggested above, participants noted ongoing practices in GH of developing policies or solutions disconnected from the needs of people on the ground. All participants highlighted the influence of GH research funders on research agenda setting, and some were quite critical. When funders prioritized their own agendas, local actors were left to adapt, owing to limited funding alternatives. The participant below explained the routine adaptation of tuberculosis research programmes and priorities within their RI to funder priorities. The power imbalance in relationships with funders, and the participant’s feelings of having no choice when priorities were imposed by funders, was quite clear:

I’m saying is that, under normal circumstances, without answering directly, I’ll take a certain funder. He’ll already put his interests first before coming and saying, he’ll say: I only work on tuberculosis. And when I work on tuberculosis, I only work on the city of [capital]. So, he arrives with his funds and tells you: I’m here and that’s that. So sometimes you take, or you don’t take, depending on … In general, we take. We adapt because we don’t have anything better. So very often the funder puts his own interests first, even when he wants to help you. We found ourselves in a situation where, I’d like to say, everyone had lost a bit: where was my interest? It was: where’s our interest? (R11, AFR)

### Theme 3: operationalizes and depends on less hierarchical ways of working

Extending the perspectives from the preceding theme, participants offered insights on how these commitments to solidarity were operationalized through, and depended on, collaboration among stakeholders and with communities. While participants primarily spoke of solidarity as a principle enacted through collaboration, their reflections pointed to different but interconnected levels of solidarity. Some participants spoke of solidarity within research teams and organizations, emphasising less hierarchical ways of working, supportive leadership and mutual accountability among colleagues. Others described solidarity across institutional and national boundaries, highlighting how funding structures and authorship practices can either reinforce or undermine equitable collaboration. A further dimension concerned solidarity with communities, grounded in aligning research priorities with those of affected populations and ensuring that knowledge production serves shared social goals.

My research agenda is always aligned, and I would say even to a greater extent, drafted by the questions I receive from the community. Because I think this is what science and what scholarship is about. It’s a scholarship that should be of benefit to others. It is not only for recreational purposes. We are not reading books for recreational purposes. We are making knowledge that helps other people. This is how I see solidarity at my level as a researcher. That you always have to help others. (R52, EMR)

Participants talked about funder-driven agendas and funding models that, in their view, limited the sorts of collaborations they regarded as central to solidarity-informed and committed research. As the following participant noted, despite ongoing calls for localization, these hierarchical practices persisted, undermining the principles of solidarity and equitable partnership in GH research:

In terms of funders and funders, I suppose it’s about where they are. Where they’re channeling their resources broadly and certainly, you know, in the international development and humanitarian sector more broadly, you know, there’s been calls for so long. To, you know, the whole localization agenda^1^ like why do we have to keep putting money through these high income-based organizations who take a big cut and control everything and channel things down to local partners eventually. (R14, SEAR)

The limitations of GH collaborations were not attributed to funding agency norms alone. Research teams and institutions were also described as sometimes inappropriately claiming “collaboration” or “partnership” with lesser resourced partners, in their GH research projects. One participant recounted an experience where researchers from certain institutions or countries were included in projects to meet funding conditions. Local partners were given minimal technical roles, such as collecting samples, while substantive analysis and decision-making took place elsewhere. The participant described such ways of working as “ticking the box” rather than genuine collaboration.

You have some funders who would impose on you the need to have collaborators from certain institutions, or certain countries, in order for you to be attractive for funding, and sometimes, depending on how this goes, you might end up, on paper, as an awardee of a grant or whatnot, but then you end up having a very meager role to play, a role, such as, maybe, aiding in collecting samples or assembling a cohort of participants, and then the samples are sent to the collaborators institution, work is done on AIDS, yes you get mentioned as an author on the paper, but then you find yourself … maybe somewhere in the middle where you can’t even use it for anything meaningful. It still happens. This, when I was an undergraduate student, I saw this happening during my undergraduate research project. It still happens now, and I think that is not true, that’s not true collaboration, that’s not solidarity, that’s just ticking the box of the funder. (R41, AFR)

Participants also discussed the role of leadership in promoting solidarity by creating enabling environments for equitable collaboration to thrive, rather than directing it. One participant described effective leadership as facilitating the expertise and power of others, building a culture in which team members feel able to act without constantly seeking permission:

Leadership is really about facilitating the power that people possess, the knowledge that they possess, to figure out ways to collaboratively, or I’ll borrow your term, to build a sense of solidarity so they know that hey, you know, if you’ve got a question you can ask me, but you know, in a really healthy organization, where would be said solidarity that, you know, you wouldn’t have to be constantly say ‘oh, well do I have permission to do this’ or ‘can I do this’, those questions are always going to come up, but it really makes a difference if people understand that leadership in this case is more a facilitating role than it is a directing, and an issuing orders kind of role. (R47, AMR)

### Theme 4: demands fair distribution of burdens and benefits in research

While the preceding theme addressed power structures and relational dynamics within the research process, this theme addresses participants’ accounts on the need for a fair distribution of research burdens and benefits. Participants shared concerns around who bears the costs of research (e.g. time, risk, labour and disruption) and who ultimately gains from it (e.g. publications, funding, capacity and health improvements). Unlike the previous theme, which focused on how researchers work together, this theme focuses on what research takes from communities and what it returns to them. Participants argued that solidarity in research requires deliberate practices that fairly distribute roles, workload, decision-making and credit, as well as benefits to avoid overburdening partners within research projects. Participants linked these practices to fostering solidarity among diverse partners and creating conditions for long-term collaborations. One participant described explicitly mapping partner roles and responsibilities, ensuring that all contributions align toward a shared goal, allowing for adjustments as projects evolve.

So, in several of the projects I’ve worked on, the big ones, whether it’s engagement with partners or those we have here in the communities, we all have different roles to play, and we work together to achieve the different goals by doing what we have. So, if I’m working on the economics side of the, the whole framework, then my goals, I will achieve my goals, but in solidarity with all the other goals that are being done. This is how our projects have always been, you know? So, when we are developing these projects, we also look at what all partners, what all partners can bring to bear and ensure that we are not overloading unnecessarily, partners with goals that they cannot achieve. So, I think all these things that we do, the process of engaging other people and ensuring that we are all on the same page is solidarity, in effects, you know, you want to make sure that you have a single goal that everyone can work towards. So, irrespective of where you are coming from, how can we collectively achieve the goal of these projects? And that’s how we’ve always worked, you know, we map out everybody’s roles and responsibilities and show that the balance of work is fair. And as we move on, if there are any areas where we think we still need to be, you know, to change or to improve, we do that, and I think that’s the way we work in many of our projects. (R43, AFR)

Other participants described the persistent inequities in the distribution of benefits, stressing the importance of recognizing the contributions of all partners. One participant shared lived experiences of researchers from the Global South contributing extensively to research projects only to be excluded from authorship or key decision-making processes. The participant linked this to the broader patterns of principal investigators from the Global North leading projects, while Global South counterparts were limited to more operational tasks.

Solidarity being, you know, in research, the communication is key. You know, needing to publish your findings, whatever you do. We also know of cases where, you know, when people are doing their papers, you know, some people when it comes to even writing, because there, there’s inadequate capacity in most of, most developing countries, I would say. You know, people will do the whole way, do everything from proposal development, collect the data, write a written address. And when it comes to writing, then we have other people coming in to write and they will find their names and will be cited in the papers. And there are people who did their job, might be left out. These are… I know there are institutions that experience that. And not to talk about even the PI, you know, in many places we have PIs having from the Global North being PIs. And then Global South people being data collectors. So, these are some of the scenarios that uh will happen where we will say it has not been, and then that comes back to my issue of not being mutual beneficial to, to the teams coming together to, to work on a specific project or activity. (R41, AFR)

In addition, participants further described how mutual benefit was often essential for sustaining collaboration. A participant (R46) explained that organizations, particularly those in low- and middle-income countries, may withdraw from research collaboration if there are no direct benefits. The participant explained that in research solidarity, it is necessary to negotiate interests and agree on shared gains from the proposal development to implementation. The participant further noted that “without a win–win situation, people have no interest to cooperate”. To ensure sustained engagement, the participant recommended offering small grants to local partners to enable data sharing and joint analysis.

Of course, there is, if like a collaborating organization not willing and no more interest, then they just, they stopped to contribute. Those kinds of things always happen. For example, the majority, especially in developing countries, each organization always asks benefit of each project. And then if we can’t provide any benefits for each collaborator, they lose interest in cooperating and communicating. And that’s very difficult, we need to negotiate with our sponsor organizations to give them a small grant. In this way, we try to communicate, for example, national statistics office, we try to give them a small grant, and then they can share their databases. Then we can analyze those data and conduct joint research in this way we are trying to cooperate. That means that each side is always seeking a win–win situation. And without a win–win situation, people have no interest to cooperate. I tried to balance, from either the international organization side, either the local side, we wanted to find one interest and try to balance everybody’s interest and find negotiation to start proposal writing. After proposal writing, we need to find funding. After funding arrives, we need to negotiate how we can implement and who will be the main collaborators, who will be main implementers. And like this way, we need to negotiate. This is all the level that solidarity requires. (R46, WPR)

### Theme 5: requires deep, active listening

Threaded throughout the interviews were expressions of commitments to understanding affected population priorities, which, as our participants repeatedly stressed, required intentionally ensuring the voices of people closest to the issues were involved and had their perspectives valued in the research they led. One participant (R42) emphasized that no research process should commence without community engagement at the earliest stages of research. The participant highlighted that communities should not only participate in studies but also help to shape the research questions themselves. For this participant, research should emerge from within the communities themselves.

The first thing would be community engagement or community involvement in the development of research ideas. They would be able to formulate questions or pose questions to their communities, which are important to them within the community and not necessarily from the researcher (R42, AFR)

Another participant expressed concern that GH initiatives often fail because they are not grounded in genuine understanding of community needs. Going further, the participant argued that solidarity and health equity depended on listening to and engaging with affected communities to ensure their voices are “within the room”.

Sometimes in the GH world, we do create policies, or we do create solutions, but not based on the needs of the people on the ground or not understanding the needs of the people on the ground. You know, so understanding the communities and how they work and their needs and how you can engage them. For me, I think that’s where when you’re thinking even about solidarity and working together in health equity, whatever have you, it must be based on understanding, what are the needs. … So again, it’s bringing the voices to those people who are really affected within the room. (R32, EUR)

Inclusion of diverse voices and creating opportunities for those voices to be heard and reflected in research, constituted one way in which listening was discussed by participants as important to bringing affected voices into decision-making spaces, and to solidarity-informed research practice. At least one participant, however, was more explicit in spelling out the relationship between listening and solidarity. Echoing all participants’ insistence on the importance of measurable, visible outcomes to solidarity efforts in GH research, that participant insisted that listening within a solidarity-informed model of GH research must be coupled with translation of what is heard into action. One participant (R28) described active efforts in their work to engage experts from diverse regions, particularly those from underrepresented continents, over recent years.

We’re trying hard to include more diversity in the researchers that we are talking with, experts from different continents, especially in the past three years. It’s really, we’re really trying to develop it. (R28, EUR)

Listening actively, intentionally, as opposed to passively, as this participant put it, was not only important for collaboration but also for nudging people in particular positions of power and influence to understand and identify specific next steps they can take, to move team or project objectives forward.

I think that you create space to listen. So listening is a verb, and its action. It could also be perceived as passive if all you do is listen, but hopefully, that’s the part of the relationship, how you foster, you know, the relationship by listening, by creating space, and you know you collaborate, and explore the possibilities of further action, perhaps. So, what can come out of that listening may propel the person trying to stand in solidarity to become more active, and I think I witnessed that a few times. People picking up the phone, and using their power, their influence, to try to do something. (R50, AMR)

Taken together, these themes show that solidarity in GH research takes different forms, from personal care and collaboration among colleagues to organizational commitments and efforts for structural change. The findings highlight how the enactment of solidarity is shaped by context and constrained by existing hierarchies, echoing theoretical distinctions between thick (value-driven and enduring) and thin (instrumental and situational) solidarities.

## Discussion

References to solidarity in GH discussions are prevalent, yet what the term solidarity means and implies for the practice of GH research remains underexplored. This paper presents findings from interviews conducted with GH RI leaders, designed to elicit their perspectives on meanings and practices of solidarity within their GH research worlds. Inclusive of both solidarity successes and challenges, participants’ accounts provide unique insight on how solidarity may be recognized and enacted in the day-to-day work and decisions GH research projects and partnerships involve. The findings characterize solidarity as something enacted through meaningful action, nurtured through ongoing and inclusive relationships, supported by fair and transparent collaboration and anchored in thoughtful, deliberate listening to communities.

Key dimensions and variations in the practice of solidarity and tensions highlighted by our interviewees echo descriptions of solidarity in the literature, while also deepening understanding of theoretical conceptualizations by demonstrating how these can play out in research decisions and relationships. Participants’ accounts reflected both relational and structural dimensions of solidarity in GH research practice. Scholars have distinguished relational forms of solidarity rooted in reciprocity, shared vulnerability and mutual recognition from structural forms concerned with shifting institutions, policies and systems that reproduce inequity [37, 38, 65]. Participants’ accounts also suggested that solidarity might sometimes be invoked alongside related concepts such as equity, fairness, benefit-sharing and community engagement. While these concepts often overlap in practice, they reflect distinct ethical emphases. Equity and distributive justice focus primarily on fair outcomes and the allocation of resources [24, 64], while practices such as benefit-sharing and engagement describe mechanisms through which research partnerships may become more inclusive [24, 26, 65]. In contrast, participants frequently described solidarity as a relational commitment that motivates and sustains these practices [59, 60]. The participants’ reflections demonstrate how these dimensions intersect: Relational efforts (e.g. trust-building, co-design and inclusive authorship) were seen as prerequisites for structural change, while structural reforms (e.g. equitable funding models and long-term capacity strengthening) created conditions in which relational solidarity could take hold.

The findings also highlighted a tension described in the literature between solidarity as a moral commitment, grounded in ethical responsibility and collective welfare, and solidarity as instrumental collaboration shaped by external incentives, reputational gains or funding structures. Participants highlighted how instrumental pressures such as short-term funding cycles and tokenistic partnerships can dilute or distort solidaristic intentions. This interplay between moral aspiration and instrumental constraint reflects the dynamics identified in critiques of GH partnerships and the political economy of research [8, 66], illustrating how solidarity is continually negotiated within environments marked by power asymmetry, competition and transparency demands.

Following Prainsack and Buyx’s (2017) proposition that solidarity is expressed through enacted commitments at interpersonal, group and institutional levels, we saw these tiers reflected in our data. Participants described interpersonal solidarity (within teams and communities), group solidarity (among institutions and networks) and institutional solidarity (embedded in funding and policy arrangements). These forms often intersected and sometimes conflicted, illustrating the tensions between solidarity as moral commitment and solidarity as instrumental collaboration. At the same time, participants’ accounts extend this framework by emphasizing sustainability, enduring partnerships and impacts of solidarity-informed research that continue beyond individual projects or funding cycles. These dimensions are less explicitly emphasized in existing theoretical definitions, which tend to focus on the willingness to bear costs in assisting others [67].

Participants framed solidarity and sustainability as deeply intertwined in GH research collaborations. They called for research collaborations that move beyond satisfying funder requirements and endure beyond project cycles. Participants emphasized that sustainability is not only about time or continuity but also about power and ownership. Specifically, it requires asking whose capacities are strengthened, whose priorities define success and who continues to benefit when funding ends. Several participants stressed that solidarity loses meaning when projects end without plans for continuity. They highlighted sustainability strategies, such as local leadership, long-term partnerships and community capacity to maintain initiatives independently. Participants framed sustainability as both a moral and practical dimension of solidarity. Achieving it requires structural change, inclusive participation and ongoing engagement beyond short-term research projects. In this sense, sustainability involves transferring and localizing decision-making power to communities and local institutions. Doing this ensures that communities have the authority and resources to sustain outcomes on their own terms. This perspective echoes literature suggesting that equitable research partnerships generate more sustainable programmes and system changes [68, 69]. Other scholars similarly call for GH to shift focus from short-term projects to long-term projects with sustainable impact [70]. In participants’ accounts, solidarity-centred research challenges dominant understandings of impact and value in GH research. Rather than prioritizing academic recognition, solidarity-oriented research emphasizes mutual benefit and long-term capacity strengthening. From their perspective, research without meaningful local involvement often produces limited impact and undermines sustainability of outcomes. This view aligns with literature arguing that equitable partnerships generate more sustainable programmes and systemic change [68, 69]

Building on existing scholarship that recognizes solidarity as more than a moral ideal [37, 38], participants described it as a concrete, guiding principle of building sustainable and mutually beneficial relationships expressed through relational practices such as active listening, redistributing decision-making and co-designing with communities. These practices, participants proposed, should be embedded in institutional structures and sustained through long-term partnerships. While it is well established that solidarity requires conscious effort and cannot be assumed [38, 65, 71], our findings extend this understanding by showing how RI leaders seek to institutionalize solidarity through governance arrangements, leadership practices and funding strategies that enable equitable collaboration across contexts.

Participants proposed that research leadership guided by a commitment to solidarity could be recognized through specific practices related to leading and facilitating research collaborations. Solidarity-informed research leadership came across in participants’ accounts as being less about directing activities, and much more about ensuring the meaningful sharing of power and associated enabling of collective agency on teams. Participants stressed creating spaces to surface the knowledge and expertise of LMIC colleagues. Their examples of solidarity in practice included listening to and learning from communities as experts. Such accounts align with an increasing emphasis on listening and humility as vital leadership qualities in GH [32, 66]. Participants described effective leaders as serving, as opposed to directing, activities. From the perspectives of participants, reframing leadership through solidarity promoted a systemic shift in research culture. Leadership is seen less as an exercise of authority, and more as an enabler of collective agency. This approach to leadership reflects a broader shift towards power sharing in GH research. Collaborations increasingly centre local priorities, redistribute decision-making authority and challenge extractive research norms [48, 72]. By reframing leadership as facilitation rather than directing, these practices intentionally disrupt hierarchical structures within GH research. Participants’ accounts suggest that solidarity-informed leadership overlaps with broader frameworks for ethical GH research practice – it values lived experiences and local knowledge over academic credentials. This aligns with calls to actively listen to and learn from communities as experts [66]. Scholars and workshop participants also linked solidaristic actions to an ethics of restraint. This, they stressed, involves slowing down and moving at the pace of inclusion as a means of sustaining equitable partnerships [73].

Findings indicate that research solidarity remains fragile, often curtailed by funder-driven priorities, institutional hierarchies and established inequities in GH. These findings align with recent critiques that remind us that solidarity in GH cannot remain an occasional ideal. Instead, it must be institutionalized in practice [28, 66]. Participants repeatedly voiced concern that solidarity in research is often invoked as a token, a symbolic gesture that provides legitimacy but does little to shift underlying power asymmetries. The fear expressed by participants echoes a growing concern in GH ethics about performative commitments. Current literature on solidarity emphasizes its fragility when framed as symbolic rhetoric instead of transformative [66, 74]. These studies argue that although calls for solidarity are common, understanding of the concept is often vague in practice, as seen with the attempted global distribution of vaccines through COVAX [28, 66, 74]. The critique of COVAX – that the failure of COVID-19 vaccine rollout was a deliberate act and not accidental – exemplifies the gap in practice identified by participants in this paper. While solidarity is often invoked, it is poorly defined in practice. Take, for example, when high-income countries claim solidarity while engaging in deliberate hoarding of vaccines at the expense of less wealthy countries [66, 74]. These insights align with participants’ statements on the need to accompany promises of solidarity with action. Some participants pointed to solidarity as the vehicle for moral awareness and public accountability. They saw solidarity as a moral infrastructure that empowers ordinary people as agents of systemic change. Solidarity, therefore, was not seen just as feeling connected but about raising awareness and encouraging people to hold those in power to account. This finding suggests that solidarity may be advanced not only within institutions but also by cultivating broader expectations among communities. This indicates that RIs cannot view solidarity solely as an internal principle; it also requires strategic engagement with external systems of power. Despite these aspirations, participants also acknowledged structural barriers such as the routine routing of resources through institutions in high-income countries, and entrenched practices that reproduce power asymmetries and limit local agency.

Participants also described numerous inequities in research collaboration that, if left unaddressed, hollow out solidarity. These inequities included practices such as the concentration of decision-making power in high-income institutions, unequal control over research funds and tokenistic forms of partnership in which local collaborators are relegated to data collection or logistical roles. Participants reflected that such dynamics reproduce dependency and prevent genuine co-production of knowledge. In their view, solidarity requires confronting these asymmetries directly, by redistributing authority, ensuring equitable authorship and enabling locally led agendas. For example, LMIC researchers are significantly underrepresented as primary authors and in key decision-making roles. This aligns with recent literature that showed authorship inequity as a marker of unequal partnerships, undermining claims of solidarity in research [75]. Participants described research solidarity as requiring intentional acts of redistribution by way of recognition and authorship, and through shifting decision-making power. This description suggests that solidarity is less a fixed state and more a continuous practice of disrupting hierarchies that otherwise go unchallenged in GH. This means researchers from high-income countries must actively share recognition and credit, ensuring fair authorship and visibility for LMIC researchers. In addition, they must intentionally cede some decision-making power to partners who have historically been excluded from decision-making positions. Although there have been efforts to affirm the leadership of LMIC researchers such as naming LMIC investigators as principal investigators on grants or lead authors in publications, our findings suggest that these constitute only modest changes. These findings are consistent with a recent review highlighting that most research collaborations remain led and dominated by institutions in high-income countries [12].

Recent studies concur that mutual benefit and equity must be operationalized through deliberate measures such as balancing who leads activities and explicitly agreeing on equitable authorship arrangements [66, 70]. Participants’ accounts of inequitable authorship, narrow roles assigned to LMIC collaborators and funder-driven research agendas illustrate how structural inequities are normalized within research practice. Given that GH research funding continues to be controlled and dominated by high-income funders [66], participants’ concerns about resources being filtered through wealthy institutions highlight an important issue: Genuine efforts to challenge these power asymmetries must include prioritizing community-level distribution of resources (Table 2). Controlling funding also empowers funders to restrict research agendas, which participants argued limits the relevance of the research to communities. Participants suggested that funder-driven research agendas can, in turn, supersede local agendas, potentially reinforcing a system of hierarchy where LMIC researchers have little to no influence, echoing recent calls for localization of research funding decisions. Given that GH research funding continues to be controlled and dominated by high-income funders [66], participants’ concerns about resources being filtered through wealthy institutions highlight an important issue: Genuine efforts to challenge these power asymmetries must include prioritizing community-level distribution of resources.

**Table 2:** Solidarity-enabling research practices

Domain	Actionable practices
Relational practices	
Co-design of research agendas and methods	Active listening and recognition of community expertise
Shared decision-making across the research cycle	Strengthening trust, centring community priorities and ensuring research reflects local perspectives
Research leadership	
Creating formal opportunities for LMIC partners to lead	Orienting leadership towards facilitation and creating enabling environments for equitable collaboration rather than direction
Practising humility, restraint and inclusive pacing	Challenging hierarchical norms; enabling collective agency and more ethical research leadership
Authorship and recognition	
Fair distribution of intellectual credit and visibility	Implementing transparent, equitable authorship policies; ensuring that recognition aligns with contributions and fostering shared ownership
Shared leadership roles across partners	Addressing entrenched inequities; negotiating interests and agreeing deliberate practices that fairly distribute roles, workload and decision-making/benefits
Accountability and reflexivity	
Community feedback mechanisms	Embedding reflexivity statements tied to documented actions
Ongoing monitoring of how solidarity commitments shape decisions	Preventing symbolic or performative uses of solidarity; embedding accountability and continuous learning

Individual and team-level practices described by participants as essential to operationalizing solidarity in global health research

Finally, participants highlighted a critical tension, which lies in whether solidarity can be incorporated and standardized within research processes (Table 3). Participants’ criticism of the symbolic uses of solidarity is a warning that, once standardized, risks becoming another performative requirement – a so-called ticked box for funders. Yet, its formalization into standards may very well push solidarity beyond an optional commitment and towards standard practice, which participants largely acknowledge is necessary. Thus, the challenge is not whether to formalize solidarity in research but how to do so in ways that avoid reducing it to a token, eroding its impact.

**Table 3:** Institutional mechanisms to embed and sustain solidarity

Domain	Institutional mechanisms
Governance structures	
Joint decision-making and oversight bodies	Embedding solidarity in governance frameworks
Clear role allocation and accountability pathways	Institutionalizing solidarity beyond individual goodwill; providing durable structures that distribute power fairly
Funding and resource flow	
Direct funding allocation to LMIC institutions	Incorporating budget lines dedicated to partnership-building and community engagement
Support for localization of funding decisions	Addressing structural asymmetries; ensuring resources reach those closest to the research context and priorities
Partnership sustainability	
Mechanisms for maintaining partnerships between projects	Supporting long-term collaboration beyond grant cycles
Institutional facilitation of relationship-centred research	Enabling continuity, strengthening mutual benefit and countering the short-termism of project-based funding
Evaluation and impact metrics	
Weighting capacity strengthening and relational outcomes	Expanding criteria to include equity, sustainability and shared benefit
Integrating community-informed indicators	Reorienting definitions of research excellence; aligning evaluation with justice and long-term community benefit

Institutional level structures identified as necessary to support and sustain solidarity-informed global health research

## Limitations

While this paper does provide rich and novel insight on how a group of RI leaders interpret solidarity and its relevance to GH research work, it is exploratory, with associated limitations. GH research work involves many stakeholders. Our interviews were with RI leaders only and offer one piece to a large complex GH system. Further research is needed to examine how the qualities of solidarity-informed GH research identified by participants are understood elsewhere. This includes perspectives from actors in other positions within the GH research world. This study draws on 13 interviews from RI leaders situated in different countries and institutes, and working with different populations. The insights we heard are not suitable for generalizing about practices or experiences of solidarity in specific locales or in research with specific populations. The broad study’s primary data collection method was interviews, which are retrospective self-reporting of events. It is important to note the possibility of social desirability bias, given the discussions around solidarity. We recognize the potential for participants to present their practices in more favourable terms than the actual realities show. Finally, as a qualitative study, these findings, while robust, cannot be generalized across all research institutions. Rather, the value of this paper lies in its depth of insight and the interpretive connections it draws, which may resonate with or inform other contexts but should not be assumed to apply broadly.

### Implications for research institutions

Research institutions should translate the ideal of solidarity into concrete, structural practices that make it an active principle guiding research and collaboration. This requires reconfiguring governance, authorship, leadership and funding flows to dismantle entrenched hierarchies in GH research – hierarchies that, as demonstrated by our findings, undermine more equitable ways of working that solidarity both operationalizes and depends upon.

Practical measures include establishing shared governance frameworks at the outset of collaborations, ensuring equitable authorship policies and intentionally allocating budgets to support relationship-building and community engagement. This point is particularly important as our findings show that solidarity requires a fair distribution of research burdens and benefits.

Relational labour, such as community engagement, must be recognized and properly resourced.

It should not be invisibly absorbed by lower-resource partners. Moreover, institutions should encourage the adoption of solidarity reflexivity statements – not as ritualistic exercises but as accountability tools linked to material shifts in power, recognition and decision-making. Such statements create structured space for the deep, active listening that participants emphasized is critical for solidarity. They also ensure that community voices shape research priorities rather than merely inform them.

As such, research institutions cannot treat solidarity as an internal ethic alone but must see it as a principle with external accountability. If solidarity is to become sustainable and transformative, it should be institutionalized in funding structures, evaluation criteria and leadership models. Our findings highlight that solidarity-informed research must account for how relationships and impacts unfold over time. Yet current funding models, structured around discrete project cycles, work against this. Funders, therefore, should explicitly evaluate solidarity practices such as equitable leadership distribution, co-designed research agendas and community feedback loops as integral markers of research excellence. Institutions must also recognize that without deliberate redistribution of recognition and decision-making power, solidarity risks being hollowed out into performative gestures that do not generate any tangible equitable outcomes. By embedding solidarity into research governance and funding accountability, institutions can foster collaborations that are both more equitable and more sustainable, enabling GH research to align with the needs and aspirations of the communities it intends to serve. These findings support three critical areas for improvement that have been identified in the literature on how to enhance research solidarity [76–78]. First, research governance should include shared structures that clearly delineate roles. For instance, research partners at the outset of any project should establish a clear role allocation and an accountability process to communities and partners. Second, funders should be intentional about budgeting for relationship building. This means that budgets should explicitly state how much is allocated towards partnership work, such as relationship-building and community engagement. In addition, core deliverables should include reporting on how researchers achieved these objectives in the reflexive statement [76]. Third, impact criteria should be expanded and research evaluation should be recentred around justice, sustainability and shared benefit, ensuring that solidarity produces the tangible equitable outcomes it promises.

## Conclusions

Solidarity in GH research is not an optional ideal, rather, it operates as a guiding principle, shaping approaches to equity, collaboration and sustainability. Research institutions, with their unique influence, hold both the capacity and the opportunity to reimagine solidarity through reflexive, embodied and relational transformations. Yet, solidarity cannot be assumed or viewed as a fixed endpoint but as an ongoing practice that researchers must consciously engage with, renew and renegotiate. The absence of a common definition highlights its contested nature, meaning responses to solidarity are subjective and context-specific. Rather than seeing this as a limitation, our findings suggest it is precisely this openness that allows solidarity to serve as both a guiding principle and a practical tool for reshaping GH research in more just and accountable ways.

## Supplementary Information


Supplementary Material 1.

## Data Availability

Datasets generated during the study are not publicly available as they contain sensitive participant information and data that could compromise participants’ confidentiality. The de-identified datasets are available on request to the lead study principal investigator: Prof. Elysee Nouvet at enouvet@uwo.ca.

## Footnotes

1 The localization agenda refers to the global movement and policy framework aimed at shifting power, resources and decision-making from international actors in GH to local actors.
