# Supplementary material for: Instituting solidarity: the role of research institutes in advancing global health equity
Source: Health Res Policy Syst. 2026 Jun 19;24:76. doi: 10.1186/s12961-026-01500-6 (PMC13528029; doi:10.1186/s12961-026-01500-6)
Supplement: Supplementary file 1 [file 12961_2026_1500_MOESM1_ESM.docx]

**Semi-structured interview guide**

**Meanings and Practices of Solidarity in Global Health: A Qualitative Investigation**

**Interview guide Research program institute directors/staff**

**Estimated duration: 50-75 min**

**Preamble**

As you will know at this point from the consent form, our team is conducting a project exploring meanings, practices, impacts and challenges of solidarity in and for global health.

The word solidarity appears in many global health discussions and policies. It has been used to reference many reasons and ways people and groups come together to address global health challenges. A common suggestion in these discussions is that solidarity – in the form of collaboration or support between people or organizations – are in a particularly good position to advance equity and social justice in and for global health. We are conducting interviews to better understand what solidarity means and looks like in specific contexts, as well as how solidarity matters or not, in the eyes of people like yourself on the front lines of global health action and advocacy.

Ultimately, our goal is to develop a tool to help those interested in collaborating across national, financial, or socio-cultural differences on global health challenges, to do so in ways that uphold and advance commitments to justice.

Before we begin, I want to ask that you reconfirm that this interview can be recorded for transcription purposes only. If you choose not to be recorded, we will be taking detailed notes of the interview.

(a) [If participant confirms, begin recording AND ask participants to reconfirm verbal consent to participate and record.]

Thank you for confirming your consent to record. I will now begin the recording. May I ask you to reconfirm verbally that this interview can be recorded for transcription purposes only? I also ask that you confirm verbally that you have agreed to participate in this study without pressure or coercion from any of the investigators, research assistants, or anyone in authority.

(b) If a participant does not confirm consent to record. [Thank you for confirming your consent not to be recorded. Note that we will take detailed notes of the interview and share the final transcripts with you to verify accuracy.]

Could you tell me what pronouns you would like me to use during this interview?

**Icebreaker/intro**

1. Tell me about your role in X organization/program/office: what are your responsibilities and what brought you to this organization/program/office, and to this role?
2. What health issue or issues are you spending most of your time on these days?

**Core questions**

1. Solidarity as a term been used by different people in different ways around the world, and certainly there are places where it is not commonly used. Towards better understanding what this term means for you, can you tell me about a time when you yourself experienced solidarity? This could be that you received solidarity, or you acted in solidarity with someone else.

*Probe:* Is solidarity different from coming together or working together? (If yes, How so?)

*Probe:* Is solidarity always between humans in your understanding, or can it be between human and non-human entities?

1a. Are there any other words or practices that reference similar meanings or practices in the context(s) where you work?

2. Has solidarity, either its presence or its absence, played a role in any of the areas of research with which you have been engaged?

If yes: How so? Can you give me a specific example?

If no or not sure: What makes you say that?

3. (May be answered by 4)

Can you think of any failures of solidarity in or for global health research?

*If yes, Probe:* What factors contributed to this failure? What would have made this less of a failure?

*If cannot think of any, Probe:* Is the word failure too strong? Can you think of an example where commitments to working together for a better good (or substitute in their definition provided in response to previous question) were not enacted to your satisfaction?

4. Do you have any examples you could share of times you/your organization have worked in solidarity with another group? (1 or 2)

*For each example…*

4a. What was the issue and why did your organization feel compelled to respond?

*Probe/additional phrasing if needed:* For example, was there a particular history, or was there anything about the issue, those involved, the broader context, or any other factors, that might have played a role in your organization deciding to support this cause?

4b. What did acting in solidarity on this issue require of your organization, or imply for your organization?

*Probe* interviewee for qualifying descriptors on any action or stance: was that action normal, exceptional, a sacrifice, contested, slow, fast

4c. What were the factors that explained people in your organization wanting to practice solidarity in this case? For example, was there a particular history, or was there anything about the issue, those involved, the broader context, or any other factors, that might have played a role in your organization deciding to support this cause?

4d. Did working in solidarity in that case pose any challenges? Can you explain?

Reword option: Were there any barriers to your organization standing in solidarity with that organization on this issue? Did showing solidarity pose any challenges?

4e. Did working in solidarity in that case yield any benefits? Probe: for whom/what

4f. Were you satisfied with this experience of solidarity? Why or why not? Would you qualify this as an example of “good” or “best practice” in solidarity?

Probe for details: what qualifies it as good, not so good, etc. Please explain.

5. Is the practice of solidarity important to global health research? Please explain.

Probe for explanation of answer:

If important, seek to understand why solidarity is important? What does solidarity enable, or what is hindered if it is absent?

If not very important, probe to understand what other factors are more important and why, as well as limits of solidaritic practices in and for global health.

If it depends: solicit detailed explanation. Depends on what? What conditions are needed for it to be a force for change?

6. With respect to the GH work you do, are there any organization/program/office or populations or groups of actors you would like to see exhibit more commitments to solidarity?

*Probe* for explanation: Tell me more. What would more commitment to solidarity by that group look like? What or how would that help?

7. Are there any organization/program/office or groups with whom you partner - funders, community groups, research groups, aid organizations, government agencies, any partner - you see as exemplary in their practice of solidarity (doing a good job when it comes to solidarity)?

If yes, *probe*: Tell me more. Clarify (if not apparent): What is it they do well or differently? Are there any others?

If no, follow up: Are there any organization/program/offices – not partners - you would recommend global health actors learn from, if we are serious about fostering more solidarity in the sector?

8. Would you like to see more solidarity practiced within or by your own organization/program/office/program/office?

If yes: How would that translate into specific practices or other changes in your organization/program/office/program/office?

If not sure or no: Would more solidaristic practices within or by your organization/program/office/program/office help it meet any of its other commitments?

9. Imagine big players in global health – major funders, private donors, and agenda-setting bodies such as the WHO – could be evaluated for their practice of solidarity: What would you recommend evaluators look at?

10. Do you have any concerns about the use of solidarity or solidaristic languages in global health?
11. Any final thoughts?

Thank you!
